# Differential Outcomes of VATS and Open Surgery in Lung Cancer Patients with Antecedent Oncological Diagnoses

**DOI:** 10.3390/jpm13101498

**Published:** 2023-10-15

**Authors:** Bogdan Cosmin Tanase, Alin Ionut Burlacu, Claudiu Eduard Nistor, Teodor Horvat, Cristian Oancea, Monica Marc, Emanuela Tudorache, Diana Manolescu

**Affiliations:** 1 Department of Thoracic Surgery, Oncology Institute “Alexandru Trestioreanu” of Bucharest, Fundeni Street 252, 022328 Bucharest, Romania; cosmin.bogdan.tanase@gmail.com (B.C.T.); ncd58@yahoo.com (C.E.N.); thorax_scumc@yahoo.com (T.H.); 2 Center for Research and Innovation in Precision Medicine of Respiratory Diseases, “Victor Babes” University of Medicine and Pharmacy, Eftimie Murgu Square 2, 300041 Timisoara, Romania; marc.monica@umft.ro (M.M.); tudorache_emanuela@yahoo.com (E.T.); 3 Department of Radiology, “Victor Babes” University of Medicine and Pharmacy, Eftimie Murgu Square 2, 300041 Timisoara, Romania; dmanolescu@umft.ro

**Keywords:** lung cancer, pulmonary disease, video-assisted thoracoscopic surgery

## Abstract

Primary lung cancer is a devastating disease with high morbidity and mortality rates. Patients with a previous oncological history may present with multiple comorbidities, unique clinical features, and unique outcomes after surgical intervention for primary lung cancer. This study aimed to compare the clinical features and outcomes of patients with a previous oncological history who underwent video-assisted thoracoscopic surgery (VATS) or open surgery (OS) for primary lung cancer. A retrospective analysis was conducted on 84 patients with a previous oncological history who underwent surgical intervention for primary lung cancer between January 2018 and January 2023. Among them, 55 patients underwent VATS, while 29 patients underwent OS. Demographic and clinical characteristics, perioperative variables, and postoperative outcomes of the two surgical groups were collected and compared. Most of the 84 patients were women (58.4%) with a high smoking prevalence (44.1%) and a median of 32.3 packs-year. The patients’ histories were most predominant for gynecologic cancers (44.4%) and colorectal cancers (18.6%). The results showed that the VATS group had a significantly shorter median hospital stay than the OS group (6.0 days vs. 12.0 days, *p*-value < 0.001). Additionally, the VATS group had lower incidences of air leaks 24 h post-surgery (12.7% vs. 48.3%, *p*-value < 0.001) and intractable pain (3.6% vs. 17.2%, *p*-value = 0.031), as well as significantly lower operative times (270 min vs. 350 min, *p*-value = 0.046). However, there were no significant differences between the VATS and OS groups in overall survival (log-rank *p*-value = 0.447). Furthermore, although the 3-month survival was significantly higher in the VATS group (98.2% vs. 79.3%, *p*-value = 0.003), only one patient from the VATS group (1.8%) and two patients from the OS group (6.9%) were still alive five years after the intervention. In conclusion, VATS is a safe and effective surgical option for patients with a previous oncological history who require surgical intervention for primary lung cancer, with shorter operative times, shorter hospital stays, and lower rates of complications compared to those of OS patients, without compromising oncological outcomes. Nevertheless, both surgical options failed to improve the 5-year survival rate, probably due to the high prevalence of comorbidities and the burden of previous cancer in this population.

## 1. Introduction

Lung cancer is often caused by exposure to cigarette smoking, air pollution, or other environmental causes. It is one of the most prevalent forms of cancer globally and a primary cause of cancer-related fatalities [1]. According to the World Health Organization (WHO), lung cancer is the most frequently diagnosed cancer worldwide, accounting for around 12% of all newly diagnosed cancers [2]. The incidence of lung cancer varies worldwide, with the greatest rates occurring in industrialized nations such as the United States, Canada, and Europe [3]. In underdeveloped countries, such as Africa and Asia, where smoking rates are lower, the incidence of lung cancer is lower [4]. With a male-to-female ratio of around 1.5:1, males are approximately 1.5 times more likely than females to acquire lung cancer [5]. Lung cancer may appear with a range of symptoms, many of which are nonspecific and might be related to other medical disorders. Common lung cancer symptoms include a persistent cough, shortness of breath, chest discomfort, unexplained weight loss, tiredness, and wheezing [6].

Lung cancer can develop as a secondary malignancy that occurs due to prior cancer treatment or exposure to cancer-causing agents [7]. Secondary lung cancer can arise from primary malignancies, including breast, colon, and lymphoma [8,9,10]. The epidemiology of lung cancer as a secondary malignancy is complex and influenced by various factors, including the type and dose of cancer treatment and patient-related factors, such as age, gender, and smoking history [11]. Some common cancer treatments that increase the risk of secondary lung cancer include radiation therapy, chemotherapy, and targeted therapy [12,13,14]. Radiation therapy, in particular, has been linked to an increased risk of developing secondary lung cancer, especially in patients who have undergone radiation therapy to the chest area. On the other hand, lung metastases differ from a second primary lung cancer and occur in approximately 30% of all cancer patients [15]. The most common primary cancer types that spread to the lungs include breast, colorectal, and renal cell carcinoma. Another study found that up to 50% of patients with advanced breast cancer developed lung metastases during their disease [16].

Treatment options for lung cancer vary according to the histology and stage of the disease and the patient’s general condition [17]. Surgery, chemotherapy, radiation therapy, targeted therapy, and immunotherapy are the most prevalent therapies for lung cancer. Chemotherapy is often used to treat advanced-stage lung cancer or to decrease tumors before surgery, while radiation therapy may be used alone or in conjunction with other therapies [18]. Immunotherapy is a relatively new approach to lung cancer treatment that stimulates the immune system to target cancer cells; it is often used to treat lung cancer at an advanced stage, which has not responded to previous therapies [19,20]. Nevertheless, surgery is the most effective therapy for lung cancer in its early stages. Still, it may not be a choice for individuals with advanced lung cancer or those who are too ill to be operated on.

Video-assisted thoracoscopic surgery (VATS) and open surgery are two surgical approaches to treating lung cancer. Research studies have compared the outcomes of VATS and open surgery (OS) in lung cancer patients, concluding that VATS is associated with lower rates of complications, less pain, and shorter hospital stays than open surgery [21]. However, other studies have reported that VATS may be associated with a slightly higher rate of cancer recurrence in some instances [22]. Nevertheless, VATS and open surgery are effective surgical approaches for treating lung cancer. The choice of surgical technique depends on several factors, including the size and location of the tumor, the stage of cancer, the patient’s overall health, and the surgeon’s experience. While VATS offers several advantages over open surgery, it may only be suitable for some cases. 

The focus of the current study on patients with a previous oncological history is grounded in the premise that these patients can present a distinct clinical scenario in terms of perioperative care. These patients often present with a greater physical status and with more comorbidities, which can significantly affect their postoperative recovery and survival. Moreover, the burden of previous cancer could also influence their overall survival after surgery for primary lung cancer, regardless of the surgical technique used. Thus, understanding the impact of these surgical options on this specific patient population can guide clinicians in decision-making and tailor perioperative care more appropriately. Therefore, in the current study, we hypothesized that the outcomes of VATS surgery did not differ from the outcomes of open surgery in patients with second primary lung cancer. The first study objective was to describe the characteristics of patients with second primary lung cancer after a previous history of malignancy. The second objective was to compare the short-term outcomes in terms of complications during surgery and post-surgical complications, the duration of hospitalization, and the 5-year survival rate in these patients after undergoing VATS or OS.

## 2. Materials and Methods

### 2.1. Design of the Study and Ethical Framework

The study was structured as a multicentric retrospective cohort analysis and was undertaken in two major medical institutions in Romania: the clinical hospital specializing in infectious diseases and pneumology situated in Timisoara and the esteemed Oncology Institute “Alexandru Trestioreanu” located in Bucharest. This study’s methodology and approach were meticulously aligned with the principles stipulated in the Declaration of Helsinki. Moreover, the research initiative was officially approved, receiving designation as approval number 23, dated 6 February 2023.

The window for collecting data spanned a five-year period from January 2018 to January 2023. The dedicated team of researchers sourced their data from the electronic hospital database, complemented with information from tangible patient records. These records provided a comprehensive account, encompassing details of treatments administered, various medical procedures undertaken, and results from different laboratory analyses conducted on the patients. 

While it can be assumed that the benefits of VATS compared to those of open surgery should hold true for this cohort, it is also plausible that patients with a previous oncological history may respond differently to surgical stress, have different recovery trajectories, or experience unique complications related to their complex medical histories. The previous oncological treatment they underwent, such as radiotherapy, might predispose them for the formation of fibrous tissue and adhesions that can complicate VATS or open surgery. Hence, it was believed important to specifically evaluate the outcomes of VATS versus open surgery in this patient population. In order to determine which surgical method is inferior, we set the margin at a clinically meaningful difference of 10% for the durations of hospital stays, operative times, and postoperative complications.

To be eligible for this study, patients had to meet several specific criteria. They had to be aged 18 or older. It was imperative that these patients had a histological confirmation of primary lung cancer. Patients with a prior history of lung cancer were not considered. However, those with a previous oncological diagnosis of a cancer type other than lung cancer, which was either fully treated or in complete remission, were included. Regarding surgical intervention, only lobectomies were considered for comparison, to avoid the confounding effects of other surgical approaches.

The exclusion criteria comprised patients with incomplete medical documentation and those who had not given their consent, as seen from their personal paper records. Additionally, if a patient’s lung cancer diagnosis was a result of a previously existing malignancy or if the patient had been diagnosed with a second primary lung cancer, they were excluded. Other reasons for exclusion encompassed patients who had undergone pre-operative chemotherapy or radiation therapy for lung cancer, those with metastatic lung cancer or invasion into nearby organs, those necessitating a switch from VATS to open surgery during the procedure, and individuals with a past history of thoracic surgery. It is noteworthy that all VATS procedures involved multiple incisions.

**Figure 1:**
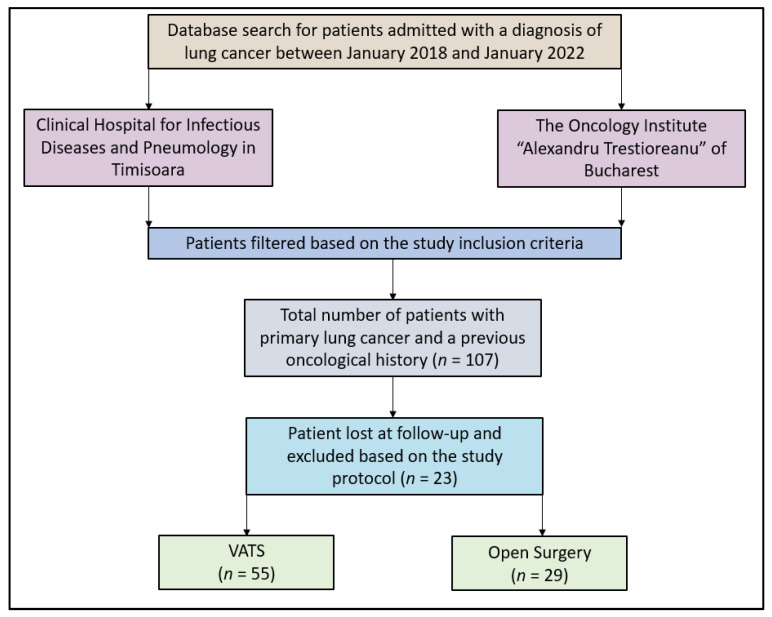
Study flowchart.

After going through the selection criteria, the entire group of patients was bifurcated. One set comprised those patients who underwent VATS, while the other consisted of patients who had open surgery. During data collection, 107 patients were pinpointed as prospective participants for the study. Nonetheless, 23 of them were ruled out due to gaps in their medical documentation, as illustrated in Figure 1. In terms of geographical distribution, two-thirds of these patients hailed from Bucharest and one-third came from Timisoara.

### 2.2. Methodology and Variables Considered

The group of participants for the study was pinpointed using the established inclusion and exclusion parameters. The basis for determining these criteria stemmed from the primary research query, ensuring that the exclusion criteria negated any potential confounding influences. Data procurement involved sifting through the medical archives of the selected group, using standardized forms for data extraction. These forms captured a spectrum of details ranging from demographics, clinical specifics, and prior treatment details to subsequent monitoring data. Once data extraction was complete, several checks were carried out to validate the accuracy and comprehensiveness of the data, ensuring that there were no missing values, unexpected outliers, or discrepancies. For subsequent in-depth analyses, these data were collated in a spreadsheet. The study incorporated a multitude of variables, including age, gender, residential location, smoking habits, exposure to respiratory threats, blood type, medical history, lung cancer specifics, duration and details of the surgical procedure, post-operative complications, and expenses.

A rigorous self-assessment, employing the Newcastle–Ottawa Scale [23], was executed to pinpoint any potential biases. The focus was on three primary biases: selection, information, and confounding factors. The study also performed a sample-size estimation to ensure sufficient statistical power, considering a significant differential of 10% in 1-year survival rates between VATS and OS groups. The AJCC guidelines and the 8th edition of the TNM system [24,25] were used to stage lung cancer in patients. Additionally, as the study was retrospective, various operators had contributed to the patient treatment.

### 2.3. Statistical Analysis

The GraphPad Prism (version 6.0) for Microsoft Windows was the chosen software for statistical analysis. The data’s normal distribution was evaluated using the Kolmogorov–Smirnov test. For data following a normal distribution, the mean and standard deviation were employed. The Student’s *t*-test was then used to examine mean variations between the comparison groups. On the other hand, for data not fitting a normal distribution, the median along with the interquartile range (IQR) was determined and showcased in box plots. The Mann–Whitney u-test was then applied to assess these variables. In situations where the chi-square test was deemed unsuitable, Fisher’s exact test was employed to compare proportions. Survival probabilities were depicted using a Kaplan–Meier curve, and a *p*-value below 0.05 was recognized as having statistical significance.

## 3. Results

### 3.1. Background Analysis

Of the 84 participants qualified for the study, 55 underwent lung cancer intervention using the VATS method and 29 underwent the open surgery route. No notable distinctions were observed in the background characteristics of these two groups. In terms of age demographics, the average age of the VATS group was slightly higher at 61.4 years, in contrast to the open surgery group’s average age of 58.3 years. The age spectrum for the participants ranged from 34 years to 84 years. A slightly higher proportion of females was evident in both groups, with the VATS and open surgery groups having 58.2% and 58.6% females, respectively. Notably, a significant portion of the study population, roughly half, either had a history of smoking or were still active smokers. The median pack-years (a measure of smoking intensity over time) for the VATS group stood at 31.5, while it was slightly higher for the open surgery group at 33.0 pack-years. However, this difference was not considered statistically significant, as detailed in Table 1.

### 3.2. Clinical and Oncological Features

**Figure 2:**
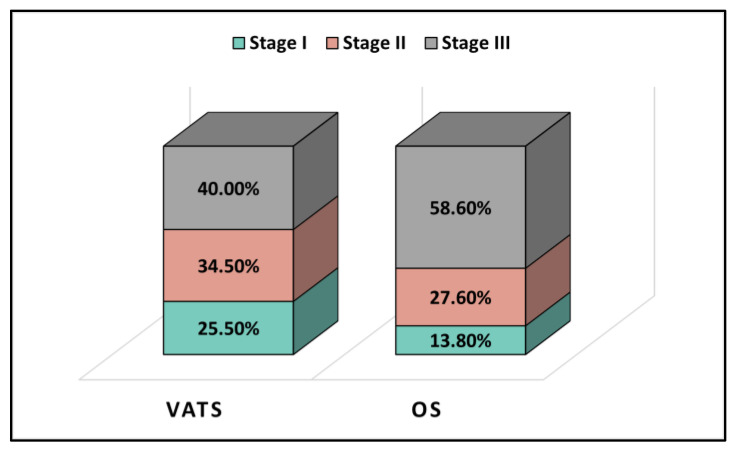
TNM cancer staging in the study cohort.

The pre-operative check showed an average FEV1% of 80.0 in the VATS group and an average FEV1% of 82.1 in the OS group, as outlined in Table 2. However, the OS group had a slightly but significantly lower left ventricle ejection fraction of 56.3%, compared to 57.7% in the VATS group (*p*-value = 0.041). The majority had NSCLC, with 85.5% in the VATS group and 93.1% in the OS group. The right lung was most affected (55.8% of all cases). The left upper lobe was the primary site in both the VATS group (29.1%) and the OS group (27.6%). As shown in Figure 2, 40.0% of VATS patients were Stage III, compared to 58.6% in the OS group.

### 3.3. Surgical Intervention and Outcomes

**Figure 3:**
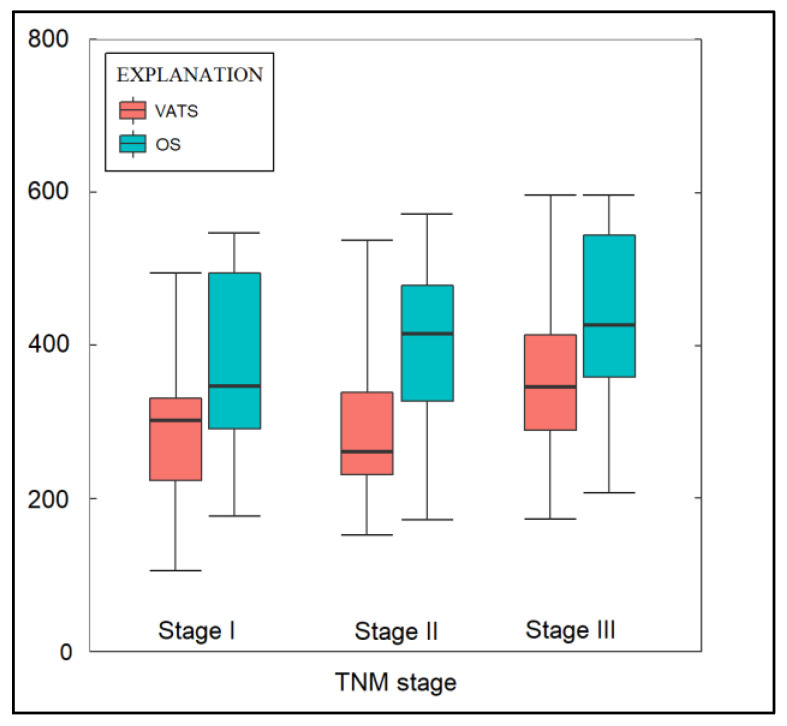
Operative time of VATS and open surgery in the study cohort, by cancer staging.

During the operation, the open surgery (OS) group experienced notably higher blood loss, with 37.9% of the patients losing over 200 mL, in contrast to just 12.8% of the patients in the VATS group (*p*-value < 0.001), as detailed in Table 3. Figure 3 shows that the OS group had a longer average operation time (350 min) than that of the VATS group (270 min), with a significant *p*-value of 0.046. While lymphadenectomy removed over two lymph node groups in 72.4% of OS cases and 65.5% in VATS, the difference was not significant. Post-operatively, the VATS group averaged a drainage of 245.9 mL within 24 h, which was significantly less than the 301.4 mL in the OS group (*p*-value < 0.001). On the second day post-op, the OS group continued to show higher drainage. The total IV fluid usage was also significantly less for the VATS group (2190 mL vs. 2551 mL in OS, *p*-value = 0.013). 

Post-operative complications indicated more frequent air leaks within the first 24 h in the OS group (48.3%) than in the VATS group (12.7%) (*p*-value < 0.001). This difference diminished a week post-op. Patients in the OS group had longer median hospital stays, both pre- and post-op (12 days post-op in OS vs. 6 days post-op in VATS, *p*-value < 0.001). However, both groups had similarly short ICU durations. Despite the longer hospital stays for OS patients, the costs of the two groups were equivalent. The Clavien–Dindo scale showed 81.8% of VATS patients and 72.4% of OS patients with scores of I or II. The rates of local and distant tumor invasion were comparable for the two groups. 

**Figure 4:**
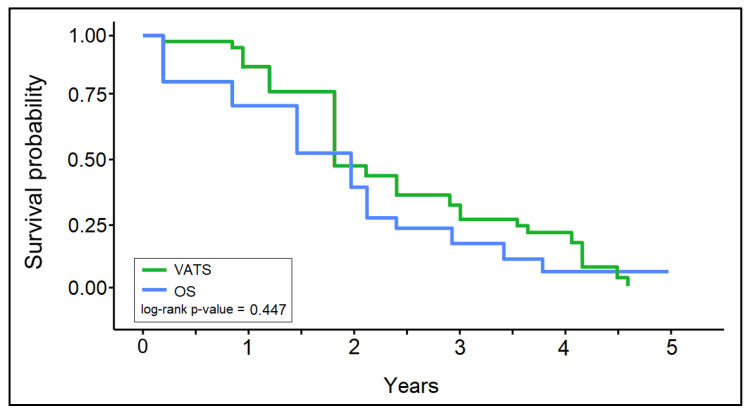
Kaplan–Meier survival curve.

The survival analysis of patients with lung cancer with a previous oncological history, presented in Table 4 and Figure 4, did not identify the survival probability (log-rank *p*-value = 0.447). However, there were significant differences in 3-month survival among patients who underwent open surgery (79.3%) compared to those who benefited from a VATS intervention (98.2%, *p*-value = 0.003), even though there was no significant difference in TNM cancer staging between the two study groups. Nevertheless, the survival at two years was low (41.8% in the VATS group and 44.8% in the OS group), although without statistical significance. Five years after the intervention, only one patient from the VATS group and two patients from the OS group (6.9%) were alive (1.8%), 

## 4. Discussion

### 4.1. Literature Findings

The current study’s novelty is its focus on a population that comprised only patients with a previous history of cancer that was cured or that was in complete remission, who developed a second primary lung cancer. The results showed that the VATS group had a significantly shorter median hospital stay and a lower incidence of air leaks 24 h post-surgery. Also, intractable pain was less common among VATS patients and VATS patients had significantly lower operative times. The two groups had no significant differences in complications, such as surgical site infections or the development of seromas. 

There were no significant differences between the VATS and OS groups in overall survival, although 3-month survival was significantly higher in the VATS group (98.2% vs. 79.3%, *p*-value = 0.003). Five years after the intervention, only one patient from the VATS group (1.8%) and two patients from the OS group (6.9%) were still alive. Thus, it was found that VATS is a safe and effective surgical option for patients with a previous oncological history who require surgical intervention for primary lung cancer, with shorter operative times, shorter hospital stays, and lower rates of complications than those of OS patients, without compromising oncological outcomes. Nevertheless, both surgical options fail to improve the 5-year survival rate, probably due to the high prevalence of comorbidities and the burden of previous cancers in this population.

All patients included in this study had a confirmed diagnosis of primary lung cancer and a previous oncological history that was in complete remission. The decision to perform VATS or open surgery was based on the treating surgeon’s professional judgement, considering the patient’s overall health, the extent and location of the tumor, and the patient’s preferences. Furthermore, in this study, there were no significant differences between the two groups in TNM staging or other comorbidities, which suggests that the surgical approach was not necessarily dictated by the severity of the disease. Also, there was a faster recovery after VATS, which might lead to a subsequent earlier initiation of adjuvant therapy that could potentially explain the higher survival rate at 3 months in the VATS group; however, our study did not collect data on the timing of adjuvant therapy initiation.

In a study by Paul S. et al., researchers compared VATS and open lobectomy in 106 patients with stage I non-small cell lung cancer (NSCLC) [26]. The study found that VATS patients had shorter hospital stays (median three days vs. five days) and less postoperative pain (visual analog scale score of 3.3 vs. 4.3) than those of open surgery patients. The two groups had no significant difference in 30-day mortality or complication. Another study published in the European Journal of Cardio–Thoracic Surgery compared VATS and open lobectomy in 220 patients with early-stage NSCLC [27]. The study found that VATS patients had shorter hospital stays (median four days vs. seven days), less blood loss (median 200 mL vs. 500 mL), and fewer complications (22.1% vs. 37.9%) than open surgery patients. There was no significant difference in 30-day mortality between the two groups. In comparison, in our study a considerable number of patients who underwent surgery had stage III NSCLC. Forty percent of patients who underwent VATS and 58.6% of patients who underwent OS were diagnosed with stage III disease. However, the standard treatment for stage III NSCLC varies across different countries and centers and is often a multimodal approach. The decision to proceed with surgery in these cases was based on multidisciplinary tumor board discussions, considering factors such as the patient’s overall health, performance status, and specific tumor characteristics.

Another study evaluated VATS and open lobectomy in 154 patients with early-stage NSCLC [28]. The study found that VATS patients had shorter hospital stays (median five days vs. eight days), lower postoperative pain (visual analog scale score of 3.8 vs. 4.9), and less blood loss (median 75 mL vs. 200 mL) than open surgery patients. The two groups had no significant difference in 30-day mortality or complication rates. Whitson et al. compared VATS and open lobectomy in 242 patients with early-stage NSCLC [29]. The study found that VATS patients had shorter hospital stays (median five days vs. eight days) and fewer complications (15.6% vs. 26.2%) than open surgery patients. The two groups had no significant difference in 30-day mortality or blood loss.

In a study published in the Annals of Thoracic Surgery, researchers compared VATS and open lobectomy in 187 patients with early-stage NSCLC. The study found that VATS patients had shorter hospital stays (median five days vs. seven days), less blood loss (median 100 mL vs. 400 mL), and fewer complications (17.2% vs. 31.9%) than open surgery patients [30]. There was no significant difference in 30-day mortality between the two groups. Another research examined the efficiency of VATS and open lobectomy in 243 patients with NSCLC. The study found that VATS patients had shorter hospital stays (median six days vs. ten days) and fewer complications (15.3% vs. 31.1%) than open surgery patients [31]. The two groups had no significant difference in 30-day mortality or blood loss. A different study indicated that VATS patients had shorter hospital stays (median six days vs. nine days) and less blood loss (median 200 mL vs. 400 mL) than open surgery patients [32]. The two groups had no significant difference in 30-day mortality or complication rates.

Another study analyzed patients who underwent VATS lobectomy and observed that they had significantly lower incidences of major complications (12.2% vs. 20.0%, *p* < 0.001) and minor complications (29.0% vs. 35.2%, *p* < 0.001) than patients who underwent open lobectomy [26]. Whitson et al. [29] conducted a retrospective analysis of 161 patients with clinical stage I non-small cell lung cancer who underwent either VATS or thoracotomy. The authors found that patients who underwent VATS had lower incidences of complications (14.2% vs. 30.2%, *p* = 0.015), shorter hospital stays (4.6 days vs. 7.6 days, *p* < 0.001), and lower 30-day mortality rates (0% vs. 5.5%, *p* = 0.042) than patients who underwent thoracotomy. A study by Onaitis et al. [30] analyzed data from 500 consecutive patients who underwent VATS lobectomy. The authors found that the overall complication rate was 15.4%, with the most common complications being atrial fibrillation (4.8%), prolonged air leaks (2.6%), and pneumonia (2.2%). The authors also reported that the median length of hospital stays was four days and the 30-day mortality rate was 0.2%. These studies suggest that VATS may be associated with lower incidences of complications and shorter hospital stays than open surgery in lung cancer patients. However, it is important to note that the surgical approach selection is based on factors such as tumor size, location, and the patient’s overall health status, and a multidisciplinary approach is required for individualized decision-making [33,34].

VATS procedures are generally reported to have shorter operative times than traditional open thoracotomy procedures. However, the specific duration of the procedure can vary, depending on the complexity of the case and the surgeon’s experience. In some studies, the operative time for VATS in primary and secondary lung cancer cases ranged from 50 min to 240 min [35]. Another study reported that VATS for secondary lung cancer had shorter operative times than those of open surgery. For instance, it was found that the mean operative time was 154.7 min for VATS and 230.3 min for open surgery [36].

The lengths of hospital stays after VATS surgery in primary and secondary lung cancer can also vary depending on the patient’s overall health and the extent of the surgery. On average, the length of hospital stays ranged from 2 to 7 days, with most patients being discharged within four days after the procedure [37]. On the other hand, patients who underwent VATS for secondary lung cancer had shorter hospital stays than those of open surgery patients. For example, a study found the median hospital stay to be four days for VATS and seven days for open surgery [38]. It can also be hypothesized that the duration of hospital stays and the patient outcomes might differ based on the type of cancer in those with a previous oncological history. Thus, in our cohort, the majority of the patients were women, and in approximately 40% of cases, they were known to have had gynecological cancer. However, other studies reported that laryngeal cancer is one of the most common types of cancer in patients with primary lung cancer [39]. Furthermore, our sample may not be fully representative of the broader lung cancer population, given the eligibility criteria and the retrospective nature of our study, as well as the relatively small sample size.

Complication rates after VATS in primary and secondary lung cancer can also vary, depending on the patient population, the extent of the surgery, and the surgeon’s experience. Some studies have reported overall complication rates ranging from 4% to 19%, with the most common complications being prolonged air leaks, pneumonia, and wound infection [40]. Several studies have reported lower overall complication rates for VATS than for open surgery for secondary lung cancer. For instance, it was found that a lower complication rate of 11.5% occurred for VATS, compared to a complication rate of 24.5% for open surgery. The most common complications reported were prolonged air leaks, wound infections, and pneumonia [26]. The mortality rate in primary and secondary lung cancer after VATS is generally low, with reported rates ranging from 0% to 3%. Mortality is more likely in patients with advanced-stage disease, poor overall health, or other comorbidities [41]. Studies have reported low mortality rates for both VATS and open surgery for secondary lung cancer. For example, a mortality rate of 0.7% was reported for VATS and 2.7% for open surgery [42].

The size of the primary or secondary lung tumors before and after the VATS procedure can be an important factor in assessing the effectiveness of the surgery. In some studies, the mean tumor size ranged from 2.1 to 5.6 cm, with smaller tumors generally being associated with better outcomes [43]. It was reported that tumor size does not significantly affect the choice of VATS versus open surgery for secondary lung cancer [44]. The frequency of cancer recurrence after VATS in primary and secondary lung cancer is an important measure of long-term success. In some studies, the recurrence rate ranged from 6% to 30%, with factors such as tumor size, stage, and location affecting the risk of recurrence [45]. Other studies have reported similar recurrence rates between VATS and open surgery for secondary lung cancer. For instance, a study found a 2-year recurrence rate of 37.5% for VATS and 43.2% for open surgery [46].

Finally, the 5-year survival rate may appear disheartening, as there were only 1.8% survivors in the VATS group after 5 years and only 6.9% survivors after 5 years in the open surgery group. Although short-term outcomes seemed improved after VATS surgery, the long-term outcomes of lung cancer patients with a previous oncological history did not change significantly. Thus, the current findings highlight the need for more comprehensive care strategies that incorporate both surgical and non-surgical treatments to optimize patient outcomes in this population.

The patient’s perceived quality of life following the VATS procedure is an important outcome measure. Some studies have reported improved quality-of-life scores after VATS in primary and secondary lung cancer cases, with patients experiencing less pain, shorter recovery times, and improved breathing function [47]. In addition, studies have reported similar or better quality of life outcomes for VATS patients than for open surgery patients with secondary lung cancer. For example, a study found better pain control and quicker recovery for VATS patients than for open surgery patients.

### 4.2. Study Strengths and Limitations

Obtaining empirical evidence to support the use of one technique over the other in specific patient populations, such as those with a previous oncological history, could provide more tailored guidance for clinicians in their surgical decision-making process. We believe that the current study adds value by filling a gap in the existing literature regarding the optimal surgical approach for primary lung cancer patients with a prior oncological history. These findings can assist in personalizing patient care, improving surgical outcomes, and enhancing the quality of life for these individuals.

Nevertheless, the current study has several limitations, including selection bias, confounding variables, limited generalizability, and lack of control. The potential for selection bias that occurs when patients are not randomly assigned to a particular treatment group was the main limitation of this study. Patients who underwent VATS or open surgery may have been selected based on age, comorbidities, and/or cancer stage, which may have resulted in a biased sample and which could have affected the validity of the study’s findings. In addition, confounding factors such as smoking history, tumor size, and histology may have confounded the relationship between the type of surgery and the outcome. However, we performed case-matching by age and gender, while the statistical analysis did not show any significant differences in the proportions of other potential confounders between the two study groups. To address this potential selection bias, a propensity score matching would be an appropriate method to use in future analyses. However, for this study, it was only possible to match patients by age and gender, while a further selection was not possible due to the retrospective nature of the study and the small sample size.

In addition, the small sample size of 84 patients in this study may limit the generalizability of the findings, since it may not be representative of the larger population of lung cancer patients. The results may not apply to patients with different characteristics or patients from different regions. Finally, retrospective cohort studies need more control of experimental designs, without which it is difficult to establish a cause-and-effect relationship between the type of surgery and the outcome. While the study identified an association between the two types of surgery, it could not determine if a type of surgery caused the outcome or whether other factors contributed to the outcome.

## 5. Conclusions

This study indicates that there is no significant difference in long-term outcomes from VATS and OS in patients with a previous oncological history. However, VATS is associated with shorter surgical lengths, shorter hospital stays, and fewer complication rates than OS, without sacrificing oncological results. Even though VATS has long-term survival rates that are comparable to those of open surgery among patients with lung cancer with a previous oncological history, neither surgical method improves the 5-year survival rate, most likely because of the high frequency of comorbidities and the burden of past malignancy in this cohort. Therefore, a closer analysis of this population is mandated to determine the risk factors that are responsible for such a low five-year survival rate.

## Figures and Tables

**Table 1:** Background data of patients treated by VATS and open surgery for lung cancer.

Variables	VATS (*n* = 55)	Open Surgery (*n* = 29)	*p*-Value
Age (mean ± SD)	61.4 ± 8.3	58.3 ± 8.4	0.108
Age range	44–84	34–73	–
Gender (male, %)	23 (41.8%)	12 (41.4%)	0.969
Area of residence (urban, %)	41 (74.5%)	24 (82.8%)	0.392
Smoking status (yes, %)	20 (36.4%)	15 (51.7%)	0.174
Pack-year smoking (median, IQR)	31.5 (24.5–38.0)	33.0 (22.0–39.5)	0.548
Exposure to respiratory hazards (yes, %)	6 (10.9%)	1 (3.4%)	0.239
Blood type A (*n*, %)	24 (43.6%)	19 (65.5%)	0.056
CCI > 3 (*n*, %)	43 (78.2%)	20 (69.0%)	0.353
**Comorbidities**			
Cardiovascular	22 (40.0%)	13 (44.8%)	0.669
Diabetes Mellitus	8 (14.5%)	3 (10.3%)	0.587
Obesity	18 (32.7%)	10 (34.5%)	0.871
CKD	5 (9.1%)	2 (6.9%)	0.729
Chronic lung disease	12 (21.8%)	7 (24.1%)	0.809
Others	4 (7.3%)	1 (3.4%)	0.481
Oncological history *			0.886
Gynecological cancer	26 (47.3%)	12 (41.4%)	
Colorectal cancer	11 (20.0%)	5 (17.2%)	
Urological cancer	8 (14.5%)	5 (17.2%)	
Others	10 (18.2%)	7 (24.1%)	

* No lung cancer history; VATS—video-assisted thoracoscopic surgery; SD—standard deviation; IQR—interquartile range; CCI—Charlson comorbidity index; CKD—chronic kidney disease.

**Table 2:** Characteristics of lung cancer in the study cohort and pre-operative findings.

Variables	VATS (*n* = 55)	Open Surgery (*n* = 29)	*p*-Value
FEV1% (mean ± SD)	80.0 ± 8.9	82.1 ± 16.4	0.447
EF% (mean ± SD)	57.7 ± 2.9	56.3 ± 3.0	0.041
**Cancer histology**			0.303
NSCLC (*n*, %)	47 (85.5%)	27 (93.1%)	
SCLC (*n*, %)	8 (14.5%)	2 (6.9%)	
**Localization**			0.916
Left lung (*n*, %)	24 (43.6%)	13 (44.8%)	
Right lung (*n*, %)	31 (56.4%)	16 (55.2%)	
**Lobe involved**			0.664
Left upper lobe	16 (29.1%)	8 (27.6%)	
Left lower lobe	8 (14.5%)	5 (17.2%)	
Right upper lobe	15 (27.3%)	6 (20.8%)	
Right middle lobe	4 (7.3%)	5 (17.2%)	
Right lower lobe	12 (21.8%)	5 (17.2%)	
**TNM classification**			0.233
Stage I (all stages)	14 (25.5%)	4 (13.8%)	
Ia	5 (9.1%)	1 (3.4%)	
Ib	9 (16.4%)	3 (10.3%)	
Stage II (all stages)	19 (34.5%)	8 (27.6%)	
IIa	11 (20.0%)	3 (10.3%)	
IIb	8 (14.5%)	5 (17.2%)	
Stage III (all stages)	22 (40.0%)	17 (58.6%)	
IIIa	14 (25.5%)	10 (34.5%)	
IIIb	8 (14.5%)	7 (24.1%)	
Grading			0.543
I	24 (43.6%)	10 (34.5%)	
II	18 (32.7%)	13 (44.8%)	
III	13 (23.6%)	6 (20.7%)	

VATS—video-assisted thoracoscopic surgery; FEV—forced expiratory Volume; EF—ejection fraction (left ventricle); NSCLC—non-small cell lung cancer; SCLC—small cell lung cancer; TNM—tumor, node, metastasis; SD—standard deviation.

**Table 3:** Surgical interventions and outcomes.

Variables	VATS (*n* = 55)	Open Surgery (*n* = 29)	*p*-Value
**Blood loss (*n*, %)**			<0.001
<100 mL	46 (83.6%)	10 (34.5%)	
100–200 mL	2 (3.6%)	8 (27.6%)	
>200 mL	7 (12.8%)	11 (37.9%)	
**Operative time, minutes (median, IQR)**	270 (220–340)	350 (300–395)	0.046
**Lymphadenectomy (*n*, %)**			0.495
1 group	6 (10.9%)	1 (3.4%)	
2 groups	13 (23.6%)	7 (24.1%)	
>2 groups	36 (65.5%)	21 (72.4%)	
**Drainage, mL (mean ± SD)**			
1st day	245.9 ± 60.3	301.4 ± 82.2	<0.001
2nd day	195.7 ± 72.4	293.3 ± 83.4	<0.001
Total intravenous fluids, mL (mean ± SD)	2190.9 ± 575.9	2551.7 ± 698.6	0.013
Local anesthesia (*n*, %)	35 (63.6%)	13 (44.8%)	0.097
Intra-op diuresis (mean ± SD)	1051.8 ± 369.6	1100 ± 329.1	0.557
Surgical site infection (*n*, %)	2 (3.6%)	3 (10.3%)	0.216
Surgical site seroma (*n*, %)	1 (1.8%)	3 (10.3%)	0.081
**Air leak**			
First day post-op	7 (12.7%)	14 (48.3%)	<0.001
1 week post-op	1 (1.8%)	2 (6.9%)	0.233
Intractable pain (*n*, %)	2 (3.6%)	5 (17.2%)	0.031
Reintervention (*n*, %)	1 (1.8%)	2 (6.9%)	0.233
**Days of hospitalization (median, IQR)**			
Before surgery	4.0 (2.0–7.0)	8.0 (3.0–11.5)	0.001
After surgery	6.0 (4.0–7.0)	12.0 (8.0–14.5)	<0.001
Days in the ICU	1.0 (1.0–1.5)	1.5 (1.0–3.0)	0.094
Clavien–Dindo score I or II, (*n*, %)	45 (81.8%)	21 (72.4%)	0.317
Local invasion after surgery (*n*, %)	4 (7.3%)	3 (17.2%)	0.160
Distant invasion after surgery (*n*, %)	5 (9.1%)	6 (20.7%)	0.134
Total expenses, RON (mean ± SD)	14,272 ± 4847	14,755 ± 9611	0.759
Adjuvant treatment	41 (74.5%)	20 (69.0%)	0.585
Radiotherapy	9 (22.0%)	5 (25.0%)	
Chemotherapy	6 (14.6%)	5 (25.0%)	
Immunotherapy	5 (12.2%)	2 (10.0%)	
Combination	21 (51.2%)	8 (40.0%)	

VATS—video-assisted thoracoscopic surgery; IQR—interquartile range; SD—standard deviation; RON—Romanian currency.

**Table 4:** Survival after VATS and open surgery.

Variables	VATS (*n* = 55)	Open Surgery (*n* = 29)	*p*-Value
1 month survival	55 (100%)	29 (100%)	-
3 months survival	54 (98.2%)	23 (79.3%)	0.003
1 year survival	42 (76.4%)	21 (72.4%)	0.691
2 years survival	23 (41.8%)	13 (44.8%)	0.791
3 years survival	12 (21.8%)	6 (20.7%)	0.904
5 years survival	1 (1.8%)	2 (6.9%)	0.233

VATS—video-assisted thoracoscopic surgery.

## Data Availability

Data are available on request.
